# One-lung ventilation to treat hepatic dome lesion – a further step towards minimally invasive surgery: a case report

**DOI:** 10.1186/s13256-019-1999-6

**Published:** 2019-03-24

**Authors:** Francesco D’Amico, Simone Serafini, Michele Finotti, Marianna Di Bello, Chiara Di Renzo, Umberto Cillo

**Affiliations:** 1 Hepatobiliary Surgery and Liver Transplantation Unit, Department of Surgery, Oncology and Gastroenterology, University Hospital of Padova, Via Giustiniani 2, 35128 Padova, Italy; 2 Department of Surgery, Division of Transplantation and Immunology, Yale University, New Haven, CT USA

**Keywords:** Minimally invasive surgery, Hepatocellular carcinoma treatment, MW ablation, One-lung ventilation, Bridge to liver transplantation

## Abstract

**Background:**

Although liver resection is still the best treatment for primary or metastatic hepatic lesions, a conventional surgical approach may be challenging in patients with a history of previous abdominal surgery. We present a case of a 58-year-old white man with paracaval, subdiaphragmatic, recurrent hepatocellular carcinoma; he had a history of multiple abdominal surgeries.

**Methods:**

In select patients, percutaneous ultrasound-guided thermal ablation is a valid non-surgical alternative due to its safety, efficacy, and good tolerability. Hepatic lesions located in the posterosuperior segments, however, can be difficult to reach via a percutaneous approach.

**Result:**

For these cases, one-lung left-sided ventilation may be particularly helpful in blocking the right hemidiaphragm and improving the acoustic window to the liver.

**Conclusion:**

We present a case of paracaval, subdiaphragmatic, recurrent hepatocellular carcinoma in which the tumor was only reachable after one-lung left-sided ventilation that was successfully treated by percutaneous ultrasound-guided microwave ablation.

## Introduction

Hepatocellular carcinoma (HCC) is one of the most common neoplasms worldwide, and liver transplantation (LT) is considered the preferred treatment of choice. However, when LT is not feasible (due to organ shortage, liver waiting list, and/or patient performance status), liver resection or locoregional treatments are the most frequent alternatives. Even if open resection has been considered the most curative strategy, in the last two decades, the efficacy of local ablation techniques has improved, especially in selected patients, showing no difference in terms of overall survival between liver resection and minimally invasive treatment [1–4].

Ultrasound (US)-guided microwave ablation (MWA) can be performed through a percutaneous, laparoscopic, transthoracic, or laparotomic approach. In very few select cases, MWA can also be used in an emergency setting, as reported recently in the literature [5]. When a laparotomic or laparoscopic approach is not feasible, a percutaneous approach represents a valid, safe, and effective alternative. However, every approach has pros and cons in relation to the clinical situation. Despite the well-known advantages of minimally invasive approaches, small and deep tumors in the posterosuperior areas of the liver, located just under the diaphragm, may not be easily achieved by a US-guided percutaneous approach [6].

## Case presentation

In August 2017, a 58-year-old white man was referred to our center of Hepatobiliary Surgery and Liver Transplantation at the Policlinic Hospital of Padua for paracaval, subdiaphragmatic recurrent HCC in the absence of underlying liver disease. He had a history of multiple abdominal surgeries: in August 2015, a laparotomic right hepatectomy for HCC (with negative oncological margins, R0); in April 2016, excision of cutaneous HCC metastases; and in January 2017, a local intrahepatic recurrence of HCC occurred, treated with liver and diaphragm en bloc resection with right diaphragmatic patch located near the resection margin. Both resections were performed in another hospital via a J-shaped incision.

**Fig. 1:**
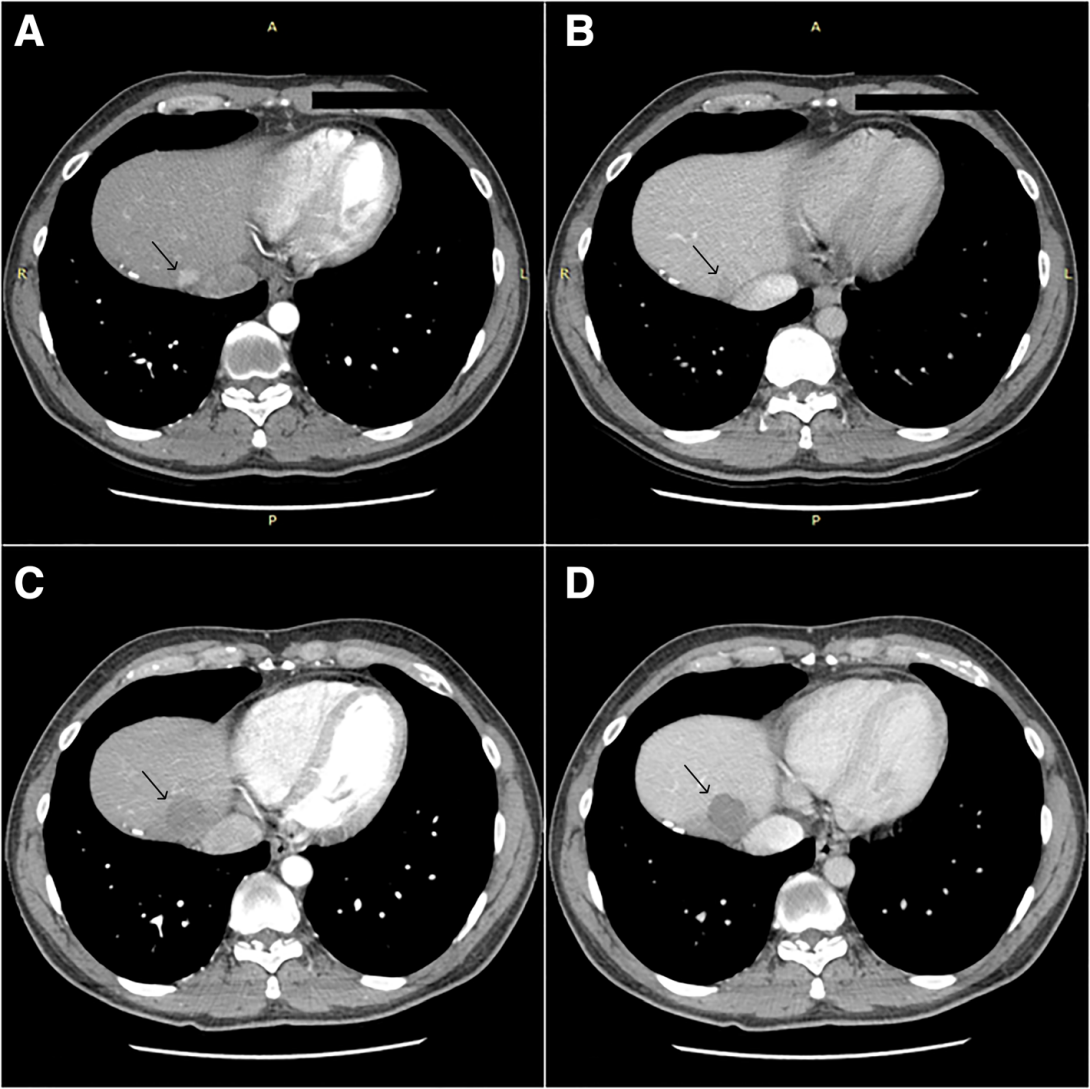
**a** and **b** Computed tomography abdominal scan, hepatocellular carcinoma nodule of 18 × 14 mm near the confluence of the middle hepatic vein and the inferior vena cava (arterial and venous phase). **c** and **d** Three-month follow-up computed tomography abdominal scan, complete necrosis of the nodule (arterial and venous phase). The area of the nodule pre-procedure and postprocedure is indicated by an *arrow*

During the follow-up, a thoracoabdominal triple-phase computed tomography (CT) scan showed a HCC nodule of 18 × 14 mm located immediately upstream of the confluence of the middle hepatic vein with the inferior vena cava (Fig. 1a, b). Abdominal US evaluation did not clearly detect the hepatic lesion due to lung and bowel interposition. He was asymptomatic, had a normal level of alpha-fetoprotein (AFP), negative hepatitis viral markers, and normal liver function: Child–Pugh A5 and Model for End-Stage Liver Disease (MELD) 6. His body mass index (BMI) was 24.

## Methods

Considering the previous multiple abdominal surgeries, initially we decided to approach the lesion thoracoscopically. This surgical procedure was performed in October 2017. Before surgery, spirometry and carbon monoxide pulmonary diffusing capacity were evaluated. A left double lumen tube was placed after induction of general anesthesia. Our patient was placed in a left lateral decubitus and mild anti-Trendelenburg position. During one-lung left-sided protective ventilation (tidal volume, 500 ml; respiratory rate, 12/minute; positive end-expiratory pressure, 6 cmH2O; fraction of inspired oxygen, 35%), the gas exchange did not differ significantly compared to that of baseline: partial pressure of oxygen in arterial blood (PaO_2_) 114 mmHg versus 135 mmHg; oxygen saturation (SatO_2_) 98.2% versus 98.4%; partial pressure of carbon dioxide in arterial blood (PaCO_2_) 38 mmHg versus 44 mmHg. The hepatic dome was scanned with a 3.5 MHz curved-array US probe through the ninth and tenth intercostal space along the right anterior axillary line. The expected hypoechogenic lesion was easily found because of the immobilization of the right-side of the diaphragm. In consideration of the better visualization of the HCC due to the lung exclusion, we changed to an intercostal percutaneous approach instead of a thoracoscopic method.

**Fig. 2:**
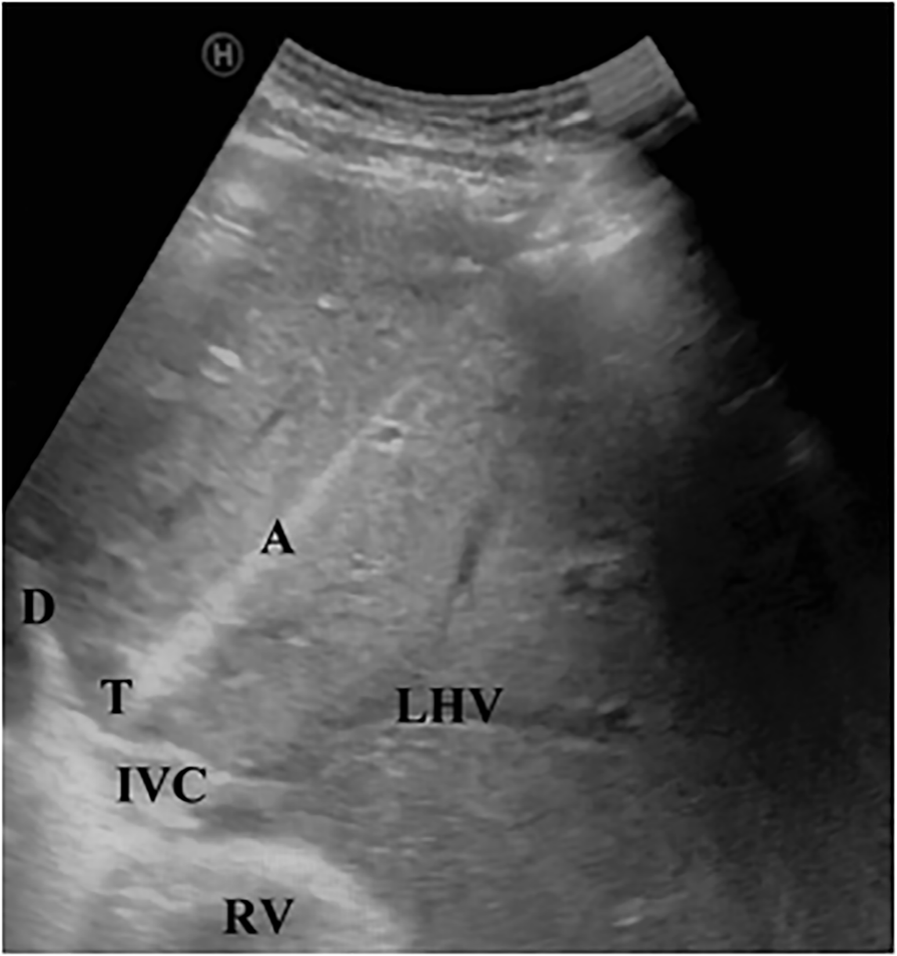
Microwave ablation needle insertion in the hepatocellular carcinoma liver nodule under ultrasound guidance. *A* antenna microwave, *D* diaphragm, *IVC* inferior vena cava, *LHV* left hepatic vein, *RV* right ventricle, *T* cancer

An internally cooled MWA antenna (AMICA generator and AMICA probe 14G, 27 mm; HS Hospital Service SpA, Aprilia, Italy; Food & Drug Administration, FDA, and Conformité Européenne, CE, approved) was inserted just above the costal margin into the lesion, with the shortest and safest needle trajectory, under real-time US guidance (Fig. 2). The probe was powered at 40 Watts for 4 minutes until the complete ablation of the lesion, including the desired safety margin.

Our patient showed stable respiratory and cardiovascular parameters during the procedure. At the end, he was quickly awakened and the double lumen tube gently removed without complications after re-expansion of his right lung. Blood count, transaminases, abdominal US examination, and chest X-ray were performed at 5 hours from the procedure to exclude early ablative complications, such as pneumothorax and unilateral pleural effusion. The postoperative period was uneventful and our patient was discharged on the same day of the procedure [7].

## Results

To evaluate the efficacy of the ablative technique, CT scans of his abdomen with contrast in arterial and portal phases were performed at 1 and 3 months post-operation. We analyzed the effect of the ablation treatment using the modified Response Evaluation Criteria in Solid Tumors (mRECIST) criteria [8]. The imaging was evaluated by three different radiologists. Safety was evaluated considering intraoperative and postoperative mortality and morbidity (30 days after surgery).

No complication occurred during or after surgery, and our patient was discharged on the day of the operation. No contrast enhancement in the target region was appreciable at the follow-up CT scans performed after 1 and 3 months, corresponding to a complete response according to mRECIST classification (Fig. 1c, d). His AFP level was also normal. Thus far, after 4 months of follow-up, no late complications have occurred.

## Conclusion

Percutaneous US-guided ablation during one-lung left-sided ventilation has proven to be clinically feasible and safe, particularly as an effective therapy for deep subdiaphragmatic hepatic lesions that are otherwise difficult to access.

Usually the right lung covers a significant portion of the upper abdomen, limiting the window of posterosuperior hepatic lesions to an intercostal percutaneous approach. To date, few reports have described percutaneous ablation with local anesthesia for HCC located in the hepatic dome close to the diaphragm [9, 10]. Even under US-guided or CT-guided ablation, especially in the presence of ascites or pleural effusion, or when the artificial type of them is locally infused to create a buffer zone, the risk of pneumothorax, burn injury, and infection remains still high [11–13].

One-lung left-sided ventilation reduces contralateral hemidiaphragm motion and improves the US window allowing easy access to the hepatic dome. General anesthesia, immobilization of the liver and real-time US permit safe ablation without risk of burn lesions to adjacent organs due to a patient’s response to pain. These features are especially helpful in cases of patient obesity, which is usually considered a contraindication to a percutaneous approach.

In patients with history of multiple prior laparotomies, in which a laparoscopic approach may be difficult to perform, this technique represents a valid alternative to a subsequent difficult and risky laparotomy or a more invasive transthoracic ablation [14–16].

The procedure is tolerable and acceptable to patients, as indicated by minimal postoperative pain and short recovery time. In comparison to the more invasive approaches it also has a lower cost and complication rate [17].

We believe the encouraging results of this case justify further investigation and clinical use of one-lung left-sided ventilation to achieve percutaneous ablation of hepatic dome tumors.

## Abbreviations

AFP: Alpha-fetoprotein
BMI: Body mass index
CE: Conformité Européenne
CT: Computed tomography
FDA: Food & Drug Administration
HCC: Hepatocellular carcinoma
LT: Liver transplantation
MELD: Model for End-Stage Liver Disease
mRECIST: Modified Response Evaluation Criteria in Solid Tumors
MWA: Microwave ablation
PaCO_2_: Partial pressure of carbon dioxide in arterial blood
PaO_2_: Partial pressure of oxygen in arterial blood
SatO_2_: Oxygen saturation
US: Ultrasound
